# Presence of Shiga toxin 2e-producing *Escherichia coli* and atypical enteropathogenic *E. coli* in an asymptomatic child

**DOI:** 10.1099/jmmcr.0.000001

**Published:** 2014-12-01

**Authors:** Miriam Rodriguez Fernandes, Aline Ignacio, Fernando H Martins, Leticia B Rocha, Roxane M. F Piazza, Tânia M. I Vaz, Mario Julio Avila-Campos, Viviane Nakano

**Affiliations:** ^1^​ Anaerobe Laboratory, Department of Microbiology, Biomedical Sciences Institute, University of São Paulo, São Paulo, SP, Brazil; ^2^​ Bacteriology Laboratory, Butantan Institute, São Paulo, Brazil; ^3^​ Bacteriology Laboratory, Adolfo Lutz Institute, São Paulo, Brazil

**Keywords:** aEPEC, asymptomatic child, diarrhoeal, STEC, *stx2e*

## Abstract

**Introduction:**

*Escherichia coli* causes gastroenteritis in humans and animals.

**Case presentation:**

In this study, both Shiga toxin-producing *E. coli* (STEC) and atypical enteropathogenic *E. coli* (EPEC) strains were identified in a stool sample from a healthy child, and they were serotyped as Shiga toxin-producing *E. coli* (STEC) ONT : H19 and atypical enteropathogenic *E. coli* (EPEC) O37 : H45.

**Conclusion:**

This is the first report, to our knowledge, of a concomitant presence of diarrhoeagenic *E. coli* (DEC) strains in an asymptomatic child. None of the microorganisms was able to produce diarrhoea, maybe because they were transient bacteria or because of the good immune status of the child. Attention should be paid to this result and it could be of interest in vaccine prospects.

## Introduction

*Escherichia coli* is the major bacterial causative agent of diarrhoea worldwide (Lanata *et al.*, 2013). Based on epidemiological and clinical features, specific virulence determinants and association with certain serotypes, diarrhoeagenic *E. coli* (DEC) can be divided into different pathotypes, enteroaggregative *E. coli* (EAEC), enterotoxigenic *E. coli* (ETEC), diffusely adherent *E. coli* (DAEC), enteroinvasive *E. coli* (EIEC), enteropathogenic *E. coli* (EPEC) and Shiga toxin-producing *E. coli* (STEC) (Croxen *et al.*, 2013).

EPEC is a significant cause of diarrhoea-associated mortality, particularly among children under five years of age (Lanata *et al.*, 2013). The central mechanism of EPEC pathogenesis is the ability to produce a characteristic histopathological lesion on the intestinal epithelium, designated attaching and effacing (A/E). This phenotype is elicited by a set of proteins encoded by genes contained in a chromosomal pathogenicity island called locus of enterocyte effacement (LEE). EPEC strains are also divided into typical (tEPEC) and atypical (aEPEC), by bundle-forming pilus (BFP) presence in tEPEC or absence in aEPEC. Epidemiological studies have demonstrated that aEPEC is more prevalent than tEPEC in both developing and developed countries, where aEPEC plays an important role as a causative agent of endemic diarrhoea and gastrointestinal outbreaks (Hernandes *et al.*, 2009).

STEC is frequently associated with gastroenteritis outbreaks with life-threatening complications, such as hemorrhagic colitis (HC) and hemolytic-uremic syndrome (HUS) (Karch *et al*., 2005). STEC produces either one or both types of Shiga toxins (Stx1 and Stx2), whose genes are encoded on prophages that are integrated in the chromosome (Schmidt, 2001). Three subtypes of Stx1 (a, c and d) and seven subtypes of Stx2 (a, b, c, d, e, f and g) have been described (Scheutz *et al.*, 2012). In addition to *stx* genes, a subset of STEC strains also carries the LEE pathogenicity island, designated enterohemorrhagic *E. coli* (EHEC), which is frequently associated with severe diseases in humans (Karch *et al.*, 2005).

Despite the epidemiological significance of EPEC and STEC as human pathogens, some individuals infected with these pathotypes show no apparent clinical signs of infection (De Moura *et al*., 2012; Nunes *et al.,* 2012). The presence of such pathogens in asymptomatic carriers is a public health concern because they act as reservoirs transmitting the disease throughout the community; however, little information is available about such cases. In this report, we describe the concomitant carriage of aEPEC and STEC by an asymptomatic child.

## Case report

Stool specimens were collected from 115 children without diarrhoea aged 3 to 12 years, who were not under antibiotic treatment. Four lactose-fermenting colonies were selected from each sample and further identified as *E. coli* by biochemical assays and PCR amplification of the 16S rRNA gene (Malinem *et al*., 2003). *E. coli* isolates were screened for the *eae* (attaching and effacing lesions), *bfpA* (bundle forming pilus), *aggR* (enteroaggregative adherence), *ipaH* (enteroinvasive mechanism), *elt* (heat-labile toxin, LT), *est* (heat-stable toxin, ST) and *stx1* and *stx2* (Shiga toxins) genes by multiplex PCR according to the protocol of Aranda *et al.* (2004).

The presence of DEC was detected in 28 (24.3 %) children, 11 (39.3 %) EAEC, 10 (35.7 %) EPEC, 5 (18 %) ETEC and 1 (3.5 %) STEC, but only one ten-year-old child harbored two pathotypes (EPEC and STEC). From the stool samples, four lactose-fermenting *E. coli* were characterized as three colonies were DEC belonging to two different pathotypes, while one was non-DEC. Two isolates showed the genotype *eae*-positive/*bfpA*-negative, and they were identified as aEPEC, and another isolate was *stx2-*positive and classified as STEC. These results were confirmed by simplex PCR using the same primers, and by sequencing of the PCR products. The STEC isolate was *stx2e*-positive as determined by PCR (Scheutz *et al.*, 2012). Serotyping of aEPEC and STEC was performed by standard procedures (Ewing, 1986). Both aEPEC isolates belonged to serotype O37 : H45, and the STEC serotype was ONT : H19. The aEPEC isolates produced intimin (Menezes *et al.*, 2009), but not BFP, as evidenced by immunoblotting (Nara *et al.*, 2010). Stx2e production was confirmed by cytotoxicity and neutralization assays on Vero cells (Mendes–Ledesma *et al.*, 2008). Table 1 summarizes the genotypic and phenotypic characteristics of aEPEC and STEC.

## Discussion

Our results show the concomitant presence of EPEC and STEC in a healthy child who was considered as an asymptomatic carrier. The presence of both bacteria was demonstrated by different techniques, including DNA sequencing, and, to our knowledge, this is the first report of the presence of both DEC in the faecal microbiota of a healthy child.

Studies have shown an association between the presence of enteric pathogens and socioeconomic, health and weather conditions, as well as different risk factors, such as inadequate hygiene, childhood habits (nail-biting and thumb-sucking) and close contact with domestic animals (dogs and cats).

Epidemiological data on occurrence, prevalence and distribution of DEC have been performed in patients with diarrhoea; however, little information is available in healthy humans without diarrhoea (Urdahl *et al.*, 2012).

STEC strain O157 : H7 is frequently observed in outbreaks of food-borne disease in humans, and STEC non-O157 has been found in sporadic cases (Reilly, 1998; Caprioli *et al.*, 1997). In this study, a non-typeable STEC, ONT : H19, was found, and it is possible that this strain cannot express its virulence factors in a healthy intestine, mainly in a child displaying good immune condition.

Bonkoungou *et al.* (2012) showed co-infections of different DEC pathotypes in children without diarrhoea, suggesting that it may be difficult to define the exact etiology of diarrhoea in children, since these pathotypes can also be found in asymptomatic children. In Brazil, the presence of EPEC in children with (15.4 %) and without (17.3 %) diarrhoea has been reported (Nunes *et al.*, 2012). EPEC is very common in children with and without diarrhoea, but the aEPEC pathotype is suggested to be commonly found in infants. Our results show the predominance of EAEC (39.3 %) and EPEC (35.7 %).

In this study, we identified the presence of two different pathotypes (aEPEC and STEC) expressing their respective *eae* and *stx* genes in a normal fecal sample of a healthy child. In addition, the presence of aEPEC O37 : H45 is rarely observed in asymptomatic humans (Sakkejha *et al*., 2013). In animals, STEC (subtype *stx*2*e*) NT : H19 is of epidemiological significance. Neither microorganism was able to produce diarrhoea in the child we studied, and this suggests other studies on the pathogenicity of DEC pathotypes in children without diarrhoea should be performed. This result suggests that both DEC can be found colonizing the intestinal ecosystem in healthy populations, and it also suggests more studies are needed to better understand their colonization or possible synergistic roles in asymptomatic populations.

The absence of data in the literature showing the presence of STEC harboring the *stx2e* gene in human intestinal microbiota makes it difficult to offer a consistent explanation of their presence in children without diarrhoea. On the other hand, the presence of both EPEC and STEC in a healthy child may represent their presence as transient bacteria, mainly STEC pathotypes that are known to cause specific diseases in animals, such as oedema in pigs and dysentery in calves.

The results suggest that STEC and EPEC can colonize the intestinal tract of healthy individuals at the same time. Thus, better understanding of how this asymptomatic colonization occurs may contribute to avoiding transmission of gastrointestinal diseases.

**Table 1.** Characteristics of aEPEC and STEC isolated from an asymptomatic child

Isolate	PCR genes	Pathotype	Serotype*	Intimin	BFP	Stx
Ec-1	*eae*	aEPEC	O37 : H45	+	−	nt
Ec-2	*eae*	aEPEC	O37 : H45	+	−	nt
Ec-3	*stx2(e)*	STEC	ONT : H19	nt	nt	+

nt, not tested.

**<?ENTCHAR ast?>:** ONT, non-typeable.

## Abbreviations

aEPEC: atypical enteropathogenic *Escherichia coli*
BFP: bundle-forming pilus
DEC: diarrhoeagenic *E. coli*
LEE: locus of enterocyte effacement
STEC: Shiga toxin-producing *E. coli*.
